# Genomic fluidity: an integrative view of gene diversity within microbial populations

**DOI:** 10.1186/1471-2164-12-32

**Published:** 2011-01-13

**Authors:** Andrey O Kislyuk, Bart Haegeman, Nicholas H Bergman, Joshua S Weitz

**Affiliations:** 1 School of Biology, Georgia Institute of Technology, Atlanta, GA 30332 USA; 2 INRIA Research Team MERE, UMR MISTEA, 34060 Montpellier, France; 3 National Biodefense Analysis and Countermeasures Center, Frederick, MD 21702, USA; 4 School of Physics, Georgia Institute of Technology, Atlanta, GA 30332 USA; 5 Current Address: Pacific Biosciences, Menlo Park, CA 94025 USA

## Abstract

**Background:**

The dual concepts of pan and core genomes have been widely adopted as means to assess the distribution of gene families within microbial species and genera. The core genome is the set of genes shared by a group of organisms; the pan genome is the set of all genes seen in any of these organisms. A variety of methods have provided drastically different estimates of the sizes of pan and core genomes from sequenced representatives of the same groups of bacteria.

**Results:**

We use a combination of mathematical, statistical and computational methods to show that current predictions of pan and core genome sizes may have no correspondence to true values. Pan and core genome size estimates are problematic because they depend on the estimation of the occurrence of rare genes and genomes, respectively, which are difficult to estimate precisely because they are rare. Instead, we introduce and evaluate a robust metric - genomic fluidity - to categorize the gene-level similarity among groups of sequenced isolates. Genomic fluidity is a measure of the dissimilarity of genomes evaluated at the gene level.

**Conclusions:**

The genomic fluidity of a population can be estimated accurately given a small number of sequenced genomes. Further, the genomic fluidity of groups of organisms can be compared robustly despite variation in algorithms used to identify genes and their homologs. As such, we recommend that genomic fluidity be used in place of pan and core genome size estimates when assessing gene diversity within genomes of a species or a group of closely related organisms.

## Background

The advent of technologies to rapidly sequence entire genomes provides a resource of sequenced genomes spanning the entire tree of life [1-4]. Indeed, as the cost and time to sequence genomes have decreased, it has become possible to sequence multiple individuals from within a species. Re-sequencing efforts have led to the following discovery: the representation of gene families in isolates from the same bacterial species is highly variable [5-9]. This variability poses conceptual as well as applied problems. Conceptually, the variability suggests the need to further re-visit species definitions that rely upon comparisons of highly conserved components of the genome, such as 16S rRNA sequences [10-14]. In addition, horizontal gene transfer and other genome rearrangements such as gene deletions and duplications can radically change the phenotype of a bacterium, even within individuals of the same species [15]. For example, the introduction of toxin genes can render a bacterium pathogenic. Hence, from an applied perspective, there is an increasing need to quantify the gene diversity of a species or genus with pathogenic potential [6,7,16-19]. The core and pan genome concepts have been proposed as a way to characterize the distribution of gene families within a group of organisms, e.g., within a species or genus [5,6,16,18,20-22]. The core genome is the set of genes found in every organism within a group (whether sequenced or not). The pan genome is the set of all genes found within organisms of a group (whether sequenced or not), including core genes and genes which appear in a fraction of genomes. Intuitively, the core genome preserves the notion that genomes of closely related organisms have something in common, while the pan genome is in accord with the finding that gene composition differs even among genomes of closely related organisms. In that sense, the core and pan genome concepts begin to address both conceptual problems (e.g., what is a bacterial species?) and applied problems (e.g., how likely is it that an individual of a given bacterial species is a pathogen?). Multiple attempts have been made to estimate the size of pan and core genomes in hopes of quantifying how open or closed a particular set of genomes is to gene exchange [5,7,8,23,24]. However, estimating the actual list of genes in the pan and core genomes remains intractable.

Thus far, attempts to quantify the size of the core and pan genomes have been based on extrapolations from a limited number of sequenced strains (usually on the order of a dozen or few dozen genomes) to the entire group (generally unknown, but easily upwards of 10^12 ^genomes). Results of such extrapolations have been widely divergent. In the most well-studied case, the pathogen *Streptococcus agalactiae*, estimates of the pan genome size vary from tens of thousands [23] to infinite [5]. Extreme variation in estimates of core and pan genome sizes makes it difficult to utilize these measures to quantify or compare the degree of acquisition and loss of gene families within a particular group or to make meaningful biological interpretations of the core and pan genome concepts. One might suspect that robust quantification of core and pan genomes sizes could be achieved with improved statistical estimation methods, combined with increased sequencing coverage. This is not the case. The problem of estimating pan and core genome sizes will not be resolved by gradual improvements in sequencing.

In this paper we demonstrate that current methods to estimate pan and core genome sizes are statistically ill-posed. We do so by demonstrating that sample gene distributions drawn from artificially generated groups of genomes with radically different pan and core genomes sizes are statistically indistinguishable. In contrast, we present an alternative diversity metric, genomic fluidity, whose expected value is equivalent whether estimated from the sample or from the true gene distribution. We then apply a bioinformatics pipeline so as to estimate genomic fluidity within 7 multiply-sequenced bacterial species containing 109 sequenced genomes. We test the robustness of genomic fluidity to changes in the number of sequenced genomes as well as to changes in alignment parameters. In so doing we demonstrate when it is possible to reliably rank order species in terms of genomic fluidity and discuss the implications of our work for inferring information about gene distributions based on subsamples.

## Results

### Pan and core genome sizes cannot be reliably estimated

**Figure 1:**
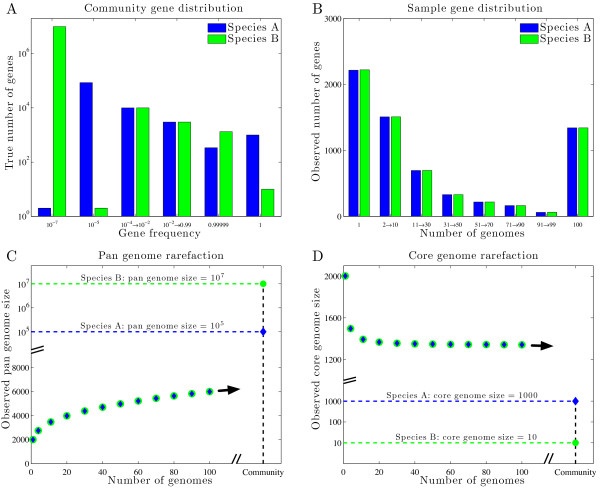
**Radically different pan and core genome sizes cannot be estimated from sampled genomes**. (A) Two species with vastly different true gene distributions: (i) Species A (blue) w/pan genome of 10^5 ^genes and core genome of 10^3 ^genes; (ii) Species B (green) w/pan genome of 10^7 ^genes and core genome of 10 genes. Each genome has 2000 genes randomly chosen from the true gene distribution according to its frequency. (B) The number of genes (y-axis) observed as a function of the number of sampled genomes (x-axis). Note that despite differences in the true distribution, the observed gene distributions are statistically indistinguishable given 100 sampled genomes. For example, there were approximately 2200 genes found in just 1 of 100 genomes for both Species A and Species B. (C) Observed pan genome size as a function of the number of sampled genomes. There is no possibility to extrapolate the true pan genome size from the observed pan genome curves. (See Additional file 1, Figure S1 for further details.) (D) Observed core genome size as a function of the number of sampled genomes. There is no possibility to extrapolate the true core genome size from the observed core genome curves.

**Figure 2:**
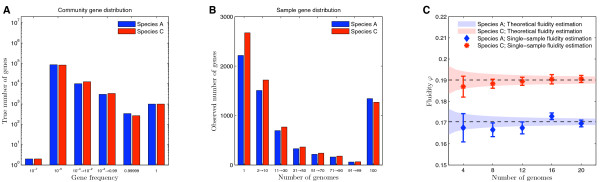
**True differences in genomic fluidity *φ *can be detected from a small number of sampled genomes**. (A) Two species with subtle differences in true gene distributions: (i) Species A (blue) as in Figure 1, w/pan genome of 10^5 ^genes and core genome of 10^3 ^genes; (ii) Species C (red) w/pan genome of 10^5 ^genes and core genome of 10^3 ^genes. Each genome has 2000 genes randomly chosen from the true gene distribution according to its frequency. (B) The number of genes (y-axis) observed as a function of the number of sampled genomes (x-axis). The observed gene distributions are statistically distinguishable. (C) Fluidity as a function of the number of sampled genomes is an unbiased estimator of the true value (dashed lines within red and blue shaded regions). The shaded regions denote the theoretical prediction for mean and standard deviations as inferred from the jackknife estimate (see Methods).

We claim that current methods to estimate pan and core genome sizes are statistically ill-posed [5,21,23,24]. To demonstrate this in a case where the pan and core genome sizes are known, we artificially generated gene distributions for three "species" such that their pan genome sizes were 10^5 ^(A), 10^7 ^(B), and 10^5 ^(C) and their core genome sizes were 10^3 ^(A), 10 (B), and 10^3 ^(C) (See Figure 1A and 2A). Note that Species A and C had distinct gene frequency distributions despite having the same pan and core genome sizes. Next, we computationally generated ensembles of genomes for each species, each of which had 2000 genes. Each gene in a genome was chosen at random from a frequency distribution specific to a given species, i.e., some genes occurred in all, or nearly all, genomes and some genes occurred very rarely. Importantly, a gene that only appears in 0.00001% of genomes (1 in 10^7 ^occurrence) contributes as much to the pan genome as does a core gene (Figure 1A), however, the rare gene will almost certainly not be detected in a sample set of tens or hundreds of sequenced genomes (Figure 1B). Furthermore, none of the genes that are detected in the sample set of genomes provide any indication that this rare gene exists while performing standard rarefaction analysis (Figure 1C). In essence, the problem of estimating the pan genome is equivalent to estimating the level of rare genes, which, because they are rare, are recalcitrant to quantification. Similar difficulties are faced when trying to quantify the size of the core genome. For example, a gene that appears in 99.999% of genomes is technically not a core gene (Figure 1A). Yet the rare genome without this core gene will not be detected in a sample set of genomes (Figure 1B), nor will the sample provide any indication that an apparent core gene is absent from some small number of organisms in the group (see Figure 1D). Intuitively, both pan and core genome size estimates depend on accurate estimation of the frequency of rare events that any small sub-sample of sequenced genomes will not enable. In principle, there may be cases where pan and core genome sizes can be accurately estimated from a subsample due to particularly low population gene diversity and/or the existence of particular parametric gene frequency distributions. However, such cases will be difficult to identify, because of the difficulty in estimating how many rare genes and rare genomes exist in the population. To further address this point, we consider alternative degrees of rarity, while continuously varying the rarest genes in the population from 10^-2 ^to less than 10^-7^. In doing so, we show that sample diversities plateau so long as the number of samples is sufficiently less than the inverse of the rarest gene (Additional file 1, Figure S1). For example, this means that one cannot estimate pan genome sizes using dozens or even hundreds of genomes if one expects that rare genes are found in one in a thousand (or less) genomes (Additional file 1, Figure S1). Hence, estimates of pan and core genome sizes may have no correspondence to true values. Despite all of these issues, the pan and core genome concepts have merit, even if their estimation is problematic. Instead, some alternative metric is needed that (i) is robust to small sample size (can be reliably estimated from few genomes); (ii) quantifies the relative degree of gene acquisition and loss within a group of genomes; and (iii) validates prior expectations that gene diversity increases within groups of increasingly unrelated organisms.

### Genomic fluidity is a robust and reliable estimator of gene diversity

We propose the use of genomic fluidity, *φ*, as a robust diversity metric which can be applied to small numbers of sequenced genomes whether at the species level or amongst groups of increasingly unrelated organisms. Genomic fluidity is defined as the ratio of unique gene families to the sum of gene families in pairs of genomes averaged over randomly chosen genome pairs from within a group of *N *genomes:

(1) $$\phi =\frac{2}{N\left(N-1\right)}\sum_{\underset{k<l}{k,l=1...N}}\frac{U_{k}+U_{l}}{M_{k}+M_{l}}.$$

where *U_k _*and *U_l _*are the number of gene families found only in genomes *k *and *l *respectively and *M_k _*and *M_l _*are the total number of gene families found in *k *and *l *respectively. Importantly, the same formula for fluidity applies whether *N *represents the total number of genomes in the population or *N *represents the total number of genomes in the sample. In other words, genomic fluidity is an estimate of gene-ic dissimilarity, akin to similarity measures used in the study of ecological communities [25] (see Additional file 1, Figure S2 for a schematic illustration of Eq. (1)). More specifically, genomic fluidity estimates how dissimilar genomes are when evaluated at a gene level. For example, a genomic fluidity of 0.1 represents that a pair of genomes have on average 10% unique genes and share 90% of their genes. As fluidity increases, so too does the probability that gene content differs between genomes in a sample. Genomic fluidity also provides information on novelty in sequencing projects. To see how, note that the best estimate for the probability that a random gene from a newly sequenced genome is not found in a randomly selected prior sequenced genome is simply *φ*. Importantly, genomic fluidity is robust to small sample size: it can be reliably estimated from a few sampled genomes. For example, in Figure 2 we show how the genomic fluidities for synthetically generated gene distributions are equivalent whether estimated from the true distribution or from a few dozen sampled genomes. In addition, subtle differences in the genomic fluidity between two species can be detected from a small number of sampled genomes. The estimated variance of fluidity was calculated using the jackknife estimate [26], which is based on leave-one-out statistics (see Methods for more details). In contrast, rarefaction curves used to estimate pan and core genome sizes are statistically indistinguishable for synthetically generated gene distributions, even when the underlying pan and core genome sizes are radically different (see Figure 1C, D).

### Fluidity and its variance can be estimated from a group of sequenced genomes

**Figure 3:**
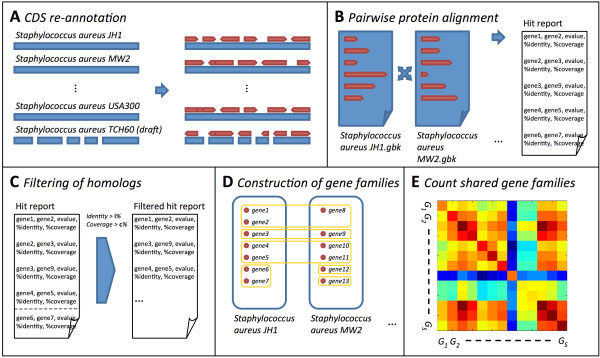
**Schematic of bioinformatics fluidity pipeline**. (A) Genomes are annotated automatically to minimize curation bias [39]; (B) For a given pair of genomes, all genes are compared using an all vs. all protein alignment; (C) Shared genes are identified based on whether alignment identity and coverage exceed i and c respectively; (D) Gene families are calculated based on a maximal clustering rule; (E) The number of shared genes is found for each pair of genomes, *G_i _*and *G_j_*, from which the number of unique genes can be calculated. Refer to the Methods for complete details of the pipeline and Additional file 1, Table S1 for a complete list of genomes analyzed.

**Figure 4:**
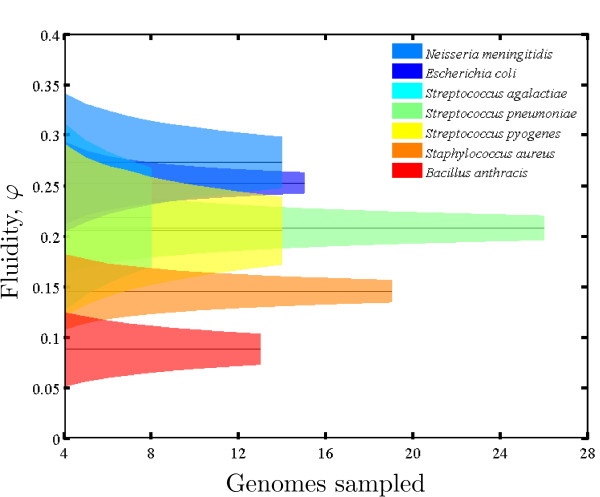
**Estimates of mean fluidity converge with increases in sampled genomes**. Fluidity was calculated as described in the text given alignment parameters *i *= 0.74 and *c *= 0.74. The variance of fluidity is estimated as a total variance, containing both the variance due to subsampling within the sample of genomes, and the variance due to the limited number of sampled genomes. For dependence of fluidity on genomes sampled for the two other sets of alignment parameters in Figure 5, see Additional file 1, Figures S3-S4.

We developed a bioinformatics pipeline to estimate genomic fluidity at the species level among sequenced genomes (see Figure 3 and Methods), but later (see Results), we apply it to more diverse groups. Using this pipeline we calculated genomic fluidity for 7 species including 109 sequenced genomes from: *Bacillus anthracis, Escherichia coli, Neisseria meningitidis, Staphylococcus aureus, Streptococcus agalactiae, Streptococcus pneumoniae*, and *Streptococcus pyogenes *(see Additional file 1, Table S1 for a list of all genomes analyzed in this study). We find that estimates of fluidity converge rapidly even when evaluated on a small number of sequenced genomes, as has been the case for all published studies of gene diversity within a species or genus. These results are consistent with the rapid convergence of fluidity when estimated from synthetically generated genomes (see Figure 2). When applied to genomes from multiply resequenced bacterial species we find the mean value of fluidity is consistent when evaluated on a small subsample or on the entire sample (Figure 4 and Additional file 1, Figures S3-S4). We find convergence of fluidity estimates to approximately 10% relative standard deviation after a dozen or so genomes (see Figure 4). The variation in fluidity estimates found in small subsamples of sequenced genomes suggests caution should be applied in attempting to establish when we can reliably say that the fluidity of a particular species is greater than that of another. Importantly, the use of the jackknife estimate of variance permits us to evaluate how both the mean and the variance of fluidity converge as more genomes are added and provides a metric to indicate when sufficient sequencing has been accomplished for use in comparing relative values of fluidity between species or between groups.

### Rank-ordering of genomic fluidity is robust to variation in alignment parameters

**Figure 5:**
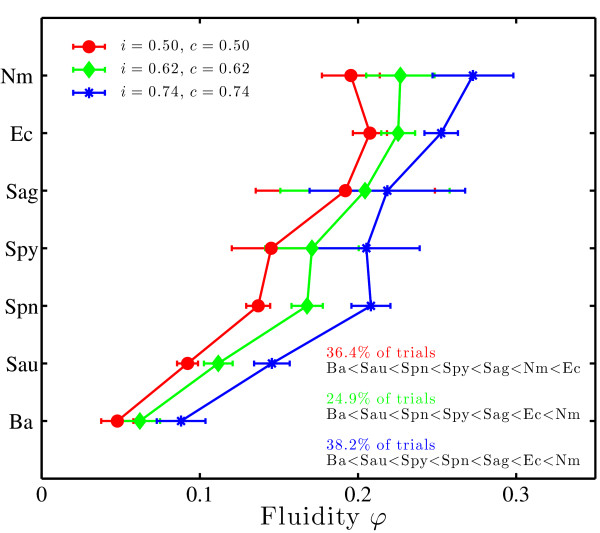
**Estimates of mean and standard deviation of fluidity for 7 multiply-sequenced species**. Mean and standard deviation of *φ *are calculated for *B*. *anthracis *(Ba), *E. coli *(Ec), and *N. meningitides *Nm). *Staph. aureus *(Sa), *Strep. agalactiae *(Sag). *Strep. pneumoniae *(Spn), and *Strep. pyogenes *(Spy) as a function of alignment parameters. Although fluidity increases with higher values of identity (*i*) and coverage (*c*) (see Additional file 1, Figure S5), only three rank-orderings of fluidity (of 5040 possible orderings) are found in 224/225 combinations of alignment parameters.

The estimate of genomic fluidity varied with alignment parameters as expected. When either minimum alignment identity or coverage is increased, more gene families are formed and fluidity increases (see Additional file 1, Figure S5). Nonetheless, the relative values of fluidity between species remained nearly invariant even as the magnitude of fluidity changed. We applied the fluidity pipeline detailed in Figure 3 and restricted our analysis to gene family assembly values of alignment identity (*i*) and coverage (*c*) from 0.5 to 0.8 in increments of 0.02 (see Methods). In 225 trials, we found 4 distinct orderings of genomic fluidity, three of which accounted for 224/225 orderings (see Figure 5 for the three dominant rank orderings). The robust rank-ordering suggests that it is possible to make comparative statements classifying one group as being more or less "open" to net gene acquisition. Specifically, we used the mean and variances estimates of fluidity to determine whether the *φ *of one species is significantly greater or less than another (see Methods). We find there is a statistically significant and unambiguous rank order of genomic fluidity for 11 = 21 comparisons of relative rank order among the 7 species examined in all 3 alignment parameter conditions corresponding to the dominant rank orderings (*p *< 0.05; see Additional file 1, Tables S2-S7). In all conditions tested, *B. anthracis *had the lowest value of *φ *and either *N*. *meningitidis *or *E. coli *had the highest value of *φ*. Further, *Strep. agalactiae *always had an intermediate value of fluidity. However, *Strep. agalactiae *had the lowest number of available genomes and a particularly high variance; therefore we were unable to rank-order it relative to any other genome with the exception of *B. anthracis*.

These results are generally consistent with previous suggestions that *B. anthracis *has a closed genome, that *N. meningitidis *may have an open genome due to its natural competence, and that *Strep. agalactiae *has an open genome [5]. However, now we can describe a group of organisms as being *relatively *open or closed, instead of being strictly open or strictly closed. In addition, we can utilize variance estimates to suggest when greater sequencing is needed. The comparison of the rank order of *φ *between species is consistent with recent calls [27] to utilize the rank, not the absolute magnitude, when comparing the relative diversity of complex ecological communities. This issue is particularly important in the case of gene diversity studies when identification of gene families is strongly depend on thresholds utilized in bioinformatics pipelines. Note that we do not suggest ranking of pan and core genome size estimates, since common genes and genomes do not, in general, inform estimates of rare genes and genomes, respectively.

### Genomic fluidity is a natural metric spanning phylogenetic scales from species to kingdom

**Figure 6:**
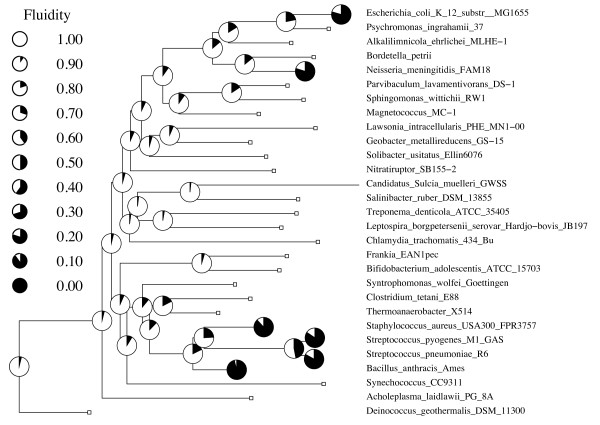
**Fluidity increases with phylogenetic scale**. Fluidity of multiply-resequenced species is in the range of 0.1 - 0.3 and the fluidity of all genomes included in the analysis approaches 1. Each circle represents the relative fluidity at a species (with multiple sequenced genomes) or internal node (the fluidity of all the genomes in the tree below it). Open circles are *φ *= 1 and black circles are *φ *= 0. The phylogenetic tree of 29 bacterial species was assembled using AMPHORA [2]. Branch lengths correspond to the average number of amino acid substitutions per position in well-conserved marker genes.

Thus far we have estimated genomic fluidity within a bacterial species, though the metric can be applied, in principle, to any group of genomes. Therefore, we estimated values of *φ *at the species level and at higher taxonomic groupings and found that *φ *varies from close to 0 (at the species level) to nearly 1 (at the phylum level) (see Figure 6). A phylogenetic tree of 29 bacterial species was assembled using AMPHORA [2]. Species in this calculation were chosen to include those whose strain-level variation we had analyzed, as well as a hand-curated selection of genomes from different parts of the tree. Each leaf with a corresponding strain group therefore represents a collapsed subtree that clusters closely around the representative strain with respect to the overall tree. The phylogenetic tree selected here is not meant to represent the entire diversity of life, but rather to illustrate how fluidity changes when closely and distantly related organisms are grouped together. Note the transition from relatively "solid" genomes at the level of isolates from within a bacterial species to a nearly totally "fluid" bacterial kingdom. Further, estimates of genomic fluidity are consistent with expectations that *φ *should increase as we move up the phylogenetic tree from species to genus to family, etc. Hence, we find that genomic fluidity is a natural metric for describing gene level similarity between groups of closely and distantly related organisms. These results suggest the suitability of genomic fluidity at coarse-grained scales, e.g. bacterial kingdom [24] and microbial community levels [28]. In contrast, estimates of pan-genome sizes at such scales will be problematic for the same reasons as outlined here when applied to closely related organisms. As a general rule, similarity based approaches to quantifying other forms of genome diversity are likely to be robust whereas estimates of the total diversity will be less so.

## Discussion

The proposal that there exists a core and pan genome for bacterial species represents a significant advance in the conceptualization of gene variability within microorganisms [5]. The basic premise of these two concepts have been borne out by the finding that the gene content of bacteria can vary significantly when comparing the sequence of two isolates from a species or genus [5-9,18,19,22]. For example, it is now well established that some genes are found in most, if not all, sequenced genomes of isolates from within a sample. In addition, it is also well established that some genes are found in very few, if only one, sequenced isolate within a sample. However, as we have demonstrated here, efforts to infer the size of the pan and core genomes of an entire species or genus from the frequency distribution of genes within a small sample of sequenced genomes will almost certainly fail. Similarly, efforts to compare the core or pan genomes sizes of bacterial species or genera will be uninformative. The reason is that pan and core genome sizes depend sensitively on the frequency of rare events (such as a rare gene occurring in a genome) whose frequency cannot be accurately estimated from a small sample of sequenced genomes. Instead, we have proposed the use of an alternative diversity metric - genomic fluidity - which is a reliable and robust estimator of the gene dissimilarity amongst a group of sequenced genomes.

This study has a number of key implications for future sequencing efforts. First, it suggests that efforts to understand a single species by sequencing as many isolates as possible may be limited in their ability to comprehensively define the diversity within that species [29]. Clearly, such studies will remain important in their ability to describe expected genomic differences (in contrast to rare genomic differences). Next, our findings also suggest that the expected gene dissimilarity within a given species can be well characterized by sequencing a relatively small number of well-chosen representatives. Sequencing a few dozen genomes is a fairly straightforward task given recent advances in sequencing technology. Finally, perhaps the most far-reaching implication of the work presented here is that we have shown it is possible to compare the relative genomic fluidity of different groups of bacteria (e.g. species, genera, or higher). We have shown that genomic fluidity can reliably distinguish between subtle differences in true gene distributions (in a computational study) as well as determine when it is possible to rank-order a set of 7 species based on the analysis of 109 whole genomes (in a bioinformatics analysis).

Genomic fluidity necessarily varies with the phylogenetic diversity of the group of genomes under consideration. In many cases, this level of diversity is defined through a species or other group definition via observed phenotypic aspects, and not through any account of genome-level divergence. As a result, within-species gene diversity of bacterial species varies greatly. To facilitate fluidity-based comparisons between species, one possibility is to normalize genomic fluidity by the average or median phylogenetic distance between members of the considered group, such as the phylogenetic distance computed using a multiple alignment of housekeeping genes [2]. However, other normalizations are possible and we consider this to be an important target for future research.

Despite its merits, genomic fluidity is not meant to describe all forms of genome variation. Genomic fluidity can provide a reliable estimate for how many new genes additional sequencing is likely to reveal, with respect to a previously sequenced genome. It cannot, however, provide an estimate of the amount of sequencing necessary to cover the gene novelty in the entire group (for reasons similar to why estimates of the pan genome size are impossible). In addition, genomic fluidity restricts itself to one component of genomic difference. There are a variety of forms of genomic differences beyond gene compositional differences or the more classic finding of single-nucleotide polymorphisms. Genomes may differ in terms of gene synteny [30], copy number variation [31,32], plasmid and/or prophage presence [17], codon biases [33,34], and methylation state [35]. It would be prudent to consider other diversity metrics, in addition to the metric of genomic fluidity studied here, that account for forms of variation in genome state amongst closely related organisms.

## Conclusions

Genomic fluidity is an integrated measure of gene diversity within a group of organisms. Genomic fluidity is both estimable given a small number of sequenced genomes and robust to variation in alignment parameters. As such, we recommend that genomic fluidity be used in place of pan and core genome size estimates when assessing gene diversity within a species or a group of closely related organisms. However, the precise relationship between variation in gene composition and genomic fluidity with underlying mechanisms of gene family diversification are yet to be resolved [15]. Recent calls for comparing and contrasting the average overlap of gene content with respect to average nucleotide divergence provide one possible route to disentangling the effects of ecological and genomic structure [36], but much work remains at the interface of bioinformatics and ecological analysis. For example, the detailed comparison of complete bacterial genomes from closely related biofilm-forming bacteria revealed how and why different organisms have adapted to and shaped their environment [37]. Similarly, genomic analysis of cyanoviruses sampled in the oceans helped uncover photosynthetic pathways which enable the exploitation of a niche distinct from previously cultured *E. coli *based phages despite sharing many common genes and genome architecture [38]. Genomic fluidity complements the detailed functional comparison of genomes by robustly estimating dissimilarity of genes within groups of genomes and providing insight into their potential evolvability. In so doing, our results highlight the need for continued focus on developing new toolsets for assessing what can be inferred about the genome composition and diversity of prokaryotic species and communities based on analysis of a sub-sample of genomes.

## Methods

### Fluidity estimator pipeline

Complete annotated genomes and draft annotated genomes were retrieved from NCBI GenBank in the GenBank format. Genomes were automatically re-annotated without hand-curation using a recently developed infrastructure resulting in new GenBank-formatted files [39]. Automatic re-annotation removes annotation bias arising from variability in annotation methods, depth of curation, and the resulting impact on the list of candidate genes - a similar approach was recently used in the analysis of genomes within a bacterial genus [18]. Following this process, putative protein sequences were extracted from annotated CDS regions and aligned using BLASTP [40] in all vs. all pairwise amino acid alignment. A pair of genes were considered homologous if the protein alignment covered more than *c *fraction of each gene's length and identity in the alignment exceeded *i*. To improve performance, alignments were parallelized between nodes on a compute cluster using the Torque PBS job scheduler.

Next, genes were clustered into gene families using a strict clique requirement, i.e. each new gene considered for inclusion into a family must have an alignment with every member of the family satisfying the minimum criteria described above. In this implementation, we compare all members of a gene family to each other on an equal basis and do not distinguish between orthologs, homologs, or paralogs. This homology-based approach is appropriate, since the fine resolution and gene family structure afforded by true ortholog reconstruction does not affect the inclusion or exclusion of genes with marginal evidence of homology.

Alignments were processed in order of increasing E-value, to prevent lower quality alignments from disrupting formation of families using higher quality alignments. Each gene was allowed to participate in only one family; if the gene could not be joined into any gene family, it formed its own singleton family. Gene family assignments were used to calculate fluidity using Eq. (1). We used the jackknife estimator [26] to estimate the variance of the fluidity estimator $\text{Var}\left[\hat{\phi }\right]$. Explicitly, for a group of *N *genomes, the variance is

(2) $${\hat{\sigma }}^{2}=\hat{Var}\left[\hat{\phi }\right]=\frac{N-1}{N}{\sum_{i}\left({\hat{\phi }}^{\left(i\right)}-\hat{\phi }\right)}^{2},$$

where ${\hat{\phi }}^{\left(i\right)}$ is the estimated fluidity based on genome pairs not including genome *i *(i.e., an estimate based on leave-one-out statistics),

(3) $${\hat{\phi }}^{\left(i\right)}=\frac{2}{\left(N-1\right)\left(N-2\right)}\sum_{\underset{k\neq i\neq l}{k<l}}\frac{U_{k}+U_{l}}{M_{k}+M_{l}}.$$

Mean and variance of fluidity for the species and conditions examined here are presented in Additional file 2.

### Significance test for fluidity differences

Consider two sets of genomes, the first set consisting of *n*_1 _genomes, the second set consisting of *n*_2 _genomes. For each pair of genomes, we determine the fraction of the total number of unique genes and the total number of genes. Averaging over all pairs in the first set gives the fluidity $\hat{\phi_{1}}$; in the second set ${\hat{\phi }}_{2}$. Suppose ${\hat{\phi }}_{1}>{\hat{\phi }}_{2}$ and we want to determine whether this inequality is significant.

From the theory of *U*-statistics it is known that the estimated fluidity has approximately a normal distribution [41]. The mean of this distribution is estimated to be ${\hat{\phi }}_{1}$ in the first set and ${\hat{\phi }}_{2}$ in the second set. The variance is estimated (by jackknifing) to be $\hat{\phi_{1}^{2}}$ in the first set and $\hat{\phi_{2}^{2}}$ in the second set. We use the parameters of the approximate normal distributions to compute the significance of the observed fluidity differences. Formally, this corresponds to a two-sample two-sided *z*-test with one degree of freedom (the effective number of degrees of freedom are taken into account by the jackknife estimation).

## Authors' contributions

AOK, BH, NHB, and JSW designed the study. AOK implemented the bioinformatics pipeline. BH and JSW developed and implemented the fluidity metric. AOK, BH and JSW analyzed results. JSW wrote the paper with the help of AOK, BH and NHB. All authors read and approved the final manuscript.

## Supplementary Material

Additional file 1 **Supplementary figures and tables**. A combined set of supplementary figures and tables referenced in the manuscript, including Figures S1-S5 and Tables S1-S7. Click here for file

Additional file 2 **Spreadsheet of fluidity values**. Mean and variance of fluidity for all species and all bioinformatic pipeline values utilized in the manuscript. Click here for file
