# Supplementary material for: Genomic fluidity: an integrative view of gene diversity within microbial populations
Source: BMC Genomics. 2011 Jan 13;12:32. doi: 10.1186/1471-2164-12-32 (PMC3030549; doi:10.1186/1471-2164-12-32)
Supplement: Additional file 1 — Supplementary figures and tables. A combined set of supplementary figures and tables referenced in the manuscript, including Figures S1-S5 and Tables S1-S7. [file 1471-2164-12-32-S1.PDF]

Supplementary File 1

Genomic fluidity: an integrative view of gene diversity within microbial populations

Andrey Kislyuk[1], Bart Haegeman[2], Nicholas H. Bergman[1,3], Joshua S. Weitz[1,4,*]

[1] *School of Biology, Georgia Institute of Technology Atlanta, GA 30332, USA.*

[2] *INRIA Research Team MERE, UMR MISTEA, 34060 Montpellier, France.*

[3] *National Biodefense Analysis and Countermeasures Center, Frederick, MD 21702, USA*

[4] *School of Physics, Georgia Institute of Technology Atlanta, GA 30332, USA.*

[*] *Corresponding author: jsweitz@gatech.edu*

January 4, 2011

Strain	Accession	CDS Original	CDS Re-annot	CDS Glimmer	CDS GeneMark	CDS BLAST
Bacillus anthracis – 13 genomes						
A0174 (Draft)	NZ_ABLT01000001	5198	5512	5641	5782	3302
A0193 (Draft)	NZ_ABKF01000001	5309	5601	5740	5889	3360
A0389 (Draft)	NZ_ABLB01000001	5296	5644	5783	5940	3398
A0442 (Draft)	NZ_ABKG01000001	5256	5598	5742	5866	3353
A0465 (Draft)	NZ_ABLH01000001	5300	5649	5782	5925	3386
A0488 (Draft)	NZ_ABJC01000001	5288	5599	5733	5900	3358
A2012 (Draft)	NZ_AAAC02000001	5352	5474	5892	5939	3341
Tsiankovskii-I (Draft)	NZ_ABDN01000001	6051	5704	5838	6025	3399
A0248 (Finished)	NC_012659	5291	5711	5855	6005	3477
AE017225 (Finished)	AE017225	5287	5427	5563	5694	3360
Ames (Finished)	NC_003997	5311	5432	5568	5695	3362
Ames_ancestor (Finished)	NC_007530	5617	5713	5856	6007	3477
CDC684 (Finished)	NC_012581	5902	5715	5859	6016	3477
Escherichia coli – 15 genomes						
536 (Finished)	NC_008253	4629	4553	4787	4699	4190
APEC01 (Finished)	NC_008563	4879	5208	5508	5385	4524
CFT073 (Finished)	NC_004431	5378	4894	5150	5055	4400
E24377A (Finished)	NC_009801	4997	4947	5174	5138	4474
EDL933 (Finished)	NC_002655	5419	5366	5744	5564	4566
HS (Finished)	NC_009800	4384	4316	4430	4449	4117
K12 (Finished)	NC_000913	4244	4320	4443	4442	4294
Sakai (Finished)	NC_002695	5341	5345	5672	5534	4563
UT189 (Finished)	NC_007946	5211	4822	5054	4979	4342
101_1 (Draft)	NZ_AAMK01000001	4234	4700	4852	4876	4334
B171 (Draft)	NZ_AAJX01000001	4705	5229	5463	5416	4574
B7A (Draft)	NZ_AAJT01000001	4628	5070	5257	5287	4494
E110019 (Draft)	NZ_AAJW01000001	4742	5239	5507	5448	4550
E22 (Draft)	NZ_AAJV01000001	4781	5328	5607	5544	4550
F11 (Draft)	NZ_AAJU01000001	4461	4884	5151	5046	4353
Neisseria meningitidis – 14 genomes						
053442 (Finished)	NC_010120	N/A	2020		Not performed	
FAM18 (Finished)	NC_008767	N/A	1918		Not performed	
MC58 (Finished)	NC_003112	N/A	2063		Not performed	
Z2491 (Finished)	NC_003116	N/A	2049		Not performed	
alpha14 (Finished)	NC_013016	N/A	2059		Not performed	
alpha153 (Draft)	N/A	N/A	2354		Not performed	

alpha275 (Draft)	N/A	N/A	2565	Not performed		
NM10699 (Draft)	N/A	N/A	2110	2494	2366	1317
NM13220 (Draft)	N/A	N/A	2299	2725	2530	1353
NM15141 (Draft)	N/A	N/A	2184	2578	2411	1369
NM15293 (Draft)	N/A	N/A	2063	2040	2062	1285
NM18575 (Draft)	N/A	N/A	2471	2927	2751	1495
NM5178 (Draft)	N/A	N/A	2097	2510	2377	1315
NM9261 (Draft)	N/A	N/A	2110	2553	2370	1341
Staphylococcus aureus – 19 genomes						
JKD6008 (Draft)	NZ_ABRZ01000084	2662	2681	2733	2791	1854
JKD6009 (Draft)	NZ_ABSA01000082	2684	2666	2720	2776	1843
MN8 (Draft)	NZ_ACJA01000014	2901	2714	2768	2845	1841
TCH60 (Draft)	NZ_ACHC01000045	2738	2551	2613	2666	1816
USA300_TCH959 (Draft)	NZ_AASB01000107	2853	2784	2826	2936	1899
COL (Finished)	NC_002951	2618	2568	2612	2680	1843
JH1 (Finished)	NC_009632	2780	2726	2775	2835	1890
JH9 (Finished)	NC_009487	2726	2726	2773	2836	1890
MRSA252 (Finished)	NC_002952	2656	2669	2728	2792	1888
MRSA_USA300_TCH1516 (Finished)	NC_010079	2689	2696	2744	2805	1890
MSSA476 (Finished)	NC_002953	2598	2555	2599	2671	1834
MW2 (Finished)	NC_003923	2632	2541	2580	2668	1832
Mu3 (Finished)	NC_009782	2698	2647	2701	2748	1876
Mu50 (Finished)	NC_002758	2731	2677	2730	2778	1885
N315 (Finished)	NC_002745	2619	2578	2624	2677	1880
NCTC8325 (Finished)	NC_007795	2892	2608	2660	2729	1830
Newman (Finished)	NC_009641	2614	2677	2722	2805	1841
RF122 (Finished)	NC_007622	2515	2589	2630	2707	1841
USA300 (Finished)	NC_007793	2604	2701	2756	2806	1884
Streptococcus agalactiae – 8 genomes						
18RS21 (Draft)	NZ_AAJO01000553	2146	2179	2326	2448	1316
515 (Draft)	NZ_AAJQ01000155	2275	2150	2248	2203	1356
COH1 (Draft)	NZ_AAJR01000393	2376	2295	2437	2341	1414
H36B (Draft)	NZ_AAJS01000345	2376	2305	2466	2354	1430
CJB111 (Draft)	NZ_AAJP01000255	2197	2099	2209	2137	1363
NEM316 (Finished)	NC_004368	2094	2127	2191	2161	1358
2603V/R (Finished)	NC_004116	2124	2108	2164	2146	1385
A909 (Finished)	NC_007432	1996	2060	2127	2094	1387
Streptococcus pneumoniae – 26 genomes						

CDC0288-04 (Draft)	NZ_ABGF01000001	1825	2015	2105	2131	1311
CDC1087-00 (Draft)	NZ_ABFT01000001	1763	2153	2230	2329	1369
CDC1873-00 (Draft)	NZ_ABFS01000001	2026	2297	2390	2464	1372
CDC3059-06 (Draft)	NZ_ABGG01000001	2088	2293	2373	2456	1327
MLV016 (Draft)	NZ_ABGH01000001	1851	2163	2253	2340	1393
SP11-BS70 (Draft)	NZ_ABAC01000001	2365	2095	2154	2221	1343
SP14-BS69 (Draft)	NZ_ABAD01000001	2807	2524	2625	2675	1461
SP18-BS74 (Draft)	NZ_ABAE01000001	2415	2144	2200	2282	1377
SP19-BS75 (Draft)	NZ_ABAF01000001	2480	2220	2300	2339	1371
SP195 (Draft)	NZ_ABGE01000001	1945	2204	2297	2353	1331
SP23-BS72 (Draft)	NZ_ABAG01000001	2416	2154	2227	2294	1337
SP3-BS71 (Draft)	NZ_AAZZ01000001	2378	2110	2191	2250	1334
SP6-BS73 (Draft)	NZ_ABAA01000001	2507	2240	2298	2373	1380
SP9-BS68 (Draft)	NZ_ABAB01000001	2429	2159	2236	2298	1336
TIGR4-454 (Draft)	NZ_AAGY02000001	1878	1994	2036	2117	1294
70585 (Finished)	NC_012468	2202	2214	2289	2340	1364
ATCC700669 (Finished)	NC_011900	1990	2195	2300	2319	1357
CGSP14 (Finished)	NC_010582	2206	2164	2231	2293	1353
D39 (Finished)	NC_008533	1914	2031	2100	2149	1306
G54_MLSTST63 (Finished)	NC_011072	2115	2085	2163	2199	1326
Hungary19A-6 (Finished)	NC_010380	2155	2249	2338	2365	1358
JJA (Finished)	NC_012466	2123	2118	2203	2247	1328
P1031 (Finished)	NC_012467	2073	2135	2221	2252	1331
R6 (Finished)	NC_003098	2043	2021	2087	2144	1301
TIGR4 (Finished)	NC_003028	2094	2139	2209	2268	1354
Taiwan19F-14 (Finished)	NC_012469	2044	2092	2158	2224	1309

Streptococcus pyogenes – 14 genomes

M49591 (Draft)	NZ_AAFV01000001	1365	1426	1457	1501	846
M1GAS (Finished)	NC_002737	1697	1791	1839	1863	1177
MGAS10270 (Finished)	NC_008022	1987	1894	1946	1976	1183
MGAS10394 (Finished)	NC_006086	1886	1826	1874	1911	1197
MGAS10750 (Finished)	NC_008024	1979	1893	1950	1972	1199
MGAS2096 (Finished)	NC_008023	1898	1813	1853	1898	1202
MGAS315 (Finished)	NC_004070	1865	1864	1920	1964	1167
MGAS5005 (Finished)	NC_007297	1865	1788	1840	1871	1187
MGAS6180 (Finished)	NC_007296	1894	1813	1871	1897	1176
MGAS8232 (Finished)	NC_003485	1845	1881	1924	1966	1191
MGAS9429 (Finished)	NC_008021	1877	1755	1800	1826	1163
Mabfredo (Finished)	NC_009332	1745	1802	1851	1893	1175
NZ131 (Finished)	NC_011375	1699	1734	1811	1817	1162

SSI-1 (Finished)	NC_004606	1861	1862	1912	1961	1167

Table S1: Accession information for all bacterial genomes used in this project. Strain lists the strain name. Accession is the NCBI accession identifier that is hyper-linked to the NCBI website. The final 5 columns denote the number of coding sequences (CDS) identified in the genome using various schemes: first, the number of CDS in the annotated genome (if available), then the number of CDS identified using the re-annotation scheme described in Materials and Methods (CDS Re-annot), and finally the number of CDS identified using Glimmer[1], GeneMarkS[2] and BLAST[3].

	Ec	Nm	Sag	Spy	Spn	Sau	Ba
Ec	×	∘	∘	⋆	⋆	⋆	⋆
Nm		×	∘	∘	⋆	⋆	⋆
Sag			×	∘	∘	∘	⋆
Spy				×	∘	⋆	⋆
Spn					×	⋆	⋆
Sau						×	⋆
Ba							×

Table S2: Significant fluidity differences for $i = 0.5$ and $c = 0.5$ (see Materials and Methods). Species are ordered such that in the upper part of the table fluidity differences are positive, e.g., *B. anthracis* (BA) has the lowest fluidity. The comparisons for which the null hypothesis that the fluidity difference is not significant can be rejected with a p-value of 0.05 are noted with a $\star$, whereas comparisons for which the null hypothesis cannot be rejected are noted with a $\circ$.

	Ec	Nm	Sag	Spy	Spn	Sau	Ba
Ec	×	$5.70\ 10^{-1}$	$7.87\ 10^{-1}$	$2.16\ 10^{-2}$	$8.57\ 10^{-8}$	$8.93\ 10^{-20}$	$5.42\ 10^{-27}$
Nm		×	$9.53\ 10^{-1}$	$1.03\ 10^{-1}$	$3.11\ 10^{-3}$	$1.14\ 10^{-7}$	$1.81\ 10^{-12}$
Sag			×	$4.49\ 10^{-1}$	$3.35\ 10^{-1}$	$8.03\ 10^{-2}$	$1.22\ 10^{-2}$
Spy				×	$7.52\ 10^{-1}$	$4.04\ 10^{-2}$	$3.04\ 10^{-4}$
Spn					×	$9.53\ 10^{-6}$	$2.44\ 10^{-12}$
Sau						×	$2.60\ 10^{-4}$
Ba							×

Table S3: p-values for fluidity differences for $i = 0.5$ and $c = 0.5$. Details of the significance test are provided in the Materials and Methods.

	Nm	Ec	Sag	Spy	Spn	Sau	Ba
Nm	$\times$	$\circ$	$\circ$	$\circ$	$\star$	$\star$	$\star$
Ec		$\times$	$\circ$	$\circ$	$\star$	$\star$	$\star$
Sag			$\times$	$\circ$	$\circ$	$\circ$	$\star$
Spy				$\times$	$\circ$	$\circ$	$\star$
Spn					$\times$	$\star$	$\star$
Sau						$\times$	$\star$
Ba							$\times$

Table S4: Significant fluidity differences for $i = 0.62$ and $c = 0.62$. Species are ordered such that in the upper part of the table fluidity differences are positive, e.g., *B. anthracis* (BA) has the lowest fluidity. The comparisons for which the null hypothesis that the fluidity difference is not significant can be rejected with a p-value of 0.05 are noted with a $\star$, whereas comparisons for which the null hypothesis cannot be rejected are noted with a $\circ$.

	Nm	Ec	Sag	Spy	Spn	Sau	Ba
Nm	$\times$	$9.53\ 10^{-1}$	$6.98\ 10^{-1}$	$1.27\ 10^{-1}$	$1.30\ 10^{-2}$	$8.98\ 10^{-7}$	$5.23\ 10^{-11}$
Ec		$\times$	$7.00\ 10^{-1}$	$8.33\ 10^{-2}$	$7.71\ 10^{-5}$	$5.66\ 10^{-16}$	$1.13\ 10^{-22}$
Sag			$\times$	$5.83\ 10^{-1}$	$5.01\ 10^{-1}$	$8.74\ 10^{-2}$	$9.66\ 10^{-3}$
Spy				$\times$	$9.22\ 10^{-1}$	$5.58\ 10^{-2}$	$7.41\ 10^{-4}$
Spn					$\times$	$2.92\ 10^{-5}$	$5.95\ 10^{-11}$
Sau						$\times$	$1.58\ 10^{-3}$
Ba							$\times$

Table S5: p-values for fluidity differences for $i = 0.62$ and $c = 0.62$. Details of the significance test are provided in the Materials and Methods.

	Nm	Ec	Sag	Spn	Spy	Sau	Ba
Nm	×	∘	∘	⋆	∘	⋆	⋆
Ec		×	∘	⋆	∘	⋆	⋆
Sag			×	∘	∘	∘	⋆
Spn				×	∘	⋆	⋆
Spy					×	∘	⋆
Sau						×	⋆
Ba							×

Table S6: Significant fluidity differences for $i = 0.74$ and $c = 0.74$. Species are ordered such that in the upper part of the table fluidity differences are positive, e.g., *B. anthracis* (BA) has the lowest fluidity. The comparisons for which the null hypothesis that the fluidity difference is not significant can be rejected with a p-value of 0.05 are noted with a $\star$, whereas comparisons for which the null hypothesis cannot be rejected are noted with a $\circ$.

	Nm	Ec	Sag	Spn	Spy	Sau	Ba
Nm	×	$4.68\ 10^{-1}$	$3.28\ 10^{-1}$	$2.30\ 10^{-2}$	$1.10\ 10^{-1}$	$5.34\ 10^{-6}$	$6.37\ 10^{-10}$
Ec		×	$4.98\ 10^{-1}$	$6.28\ 10^{-3}$	$1.80\ 10^{-1}$	$4.19\ 10^{-12}$	$1.41\ 10^{-18}$
Sag			×	$8.37\ 10^{-1}$	$8.24\ 10^{-1}$	$1.47\ 10^{-1}$	$1.13\ 10^{-2}$
Spn				×	$9.36\ 10^{-1}$	$1.76\ 10^{-4}$	$1.19\ 10^{-9}$
Spy					×	$9.21\ 10^{-2}$	$1.54\ 10^{-3}$
Sau						×	$2.60\ 10^{-3}$
Ba							×

Table S7: p-values for fluidity differences for $i = 0.74$ and $c = 0.74$. Details of the significance test are provided in the Materials and Methods.

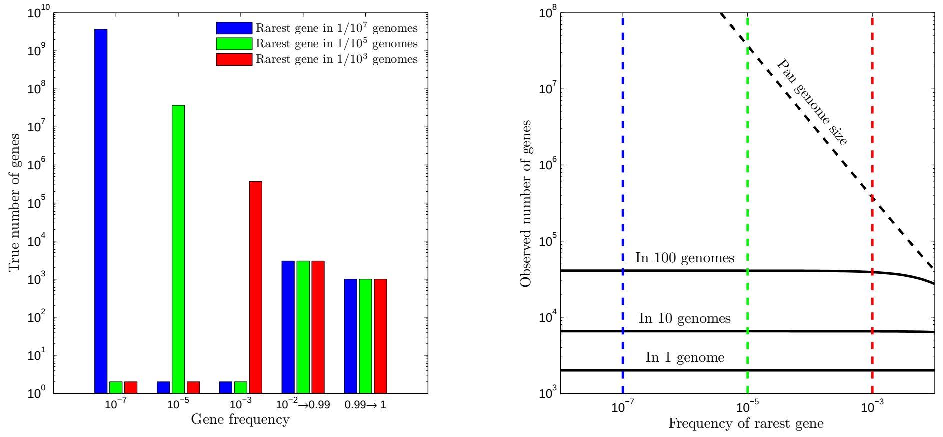

Figure S1: The impact of gene rarity on pan genome size estimation. The left-hand panel shows the gene frequency distribution of three genome models. The three models have the same common genes (gene frequency larger than 10^{-2}); they differ in the rare genes (the rare genes have frequency 10^{-7} in the blue model, 10^{-5} in the green model, and 10^{-3} in the red model). The number of rare genes is chosen such that each genome consists of 2000 genes (on average). The right-hand panel shows pan genome rarefaction data for different genome models. The genome models are listed on the X-axis. The colored dashed lines indicate the position of the three genome models of the left-hand panel. The full black line is the number of genes observed in a sample of 1 genome, 10 genomes and 100 genomes. The dashed black line is the true pan genome size of the genome model. Note that with increasing rarity, there is little to no difference in observed genes (flat black lines) and so the true pan genome size cannot be estimated (dashed black line).

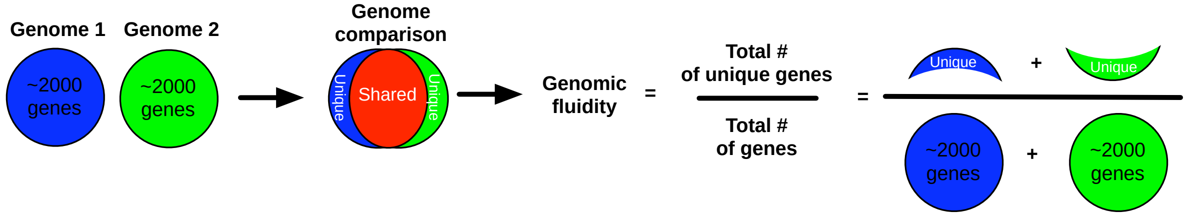

Figure S2: Schematic of Eq. (1) for calculating genomic fluidity based on tabulating the ratio of unique gene families to total gene families amongst pairs of genomes.

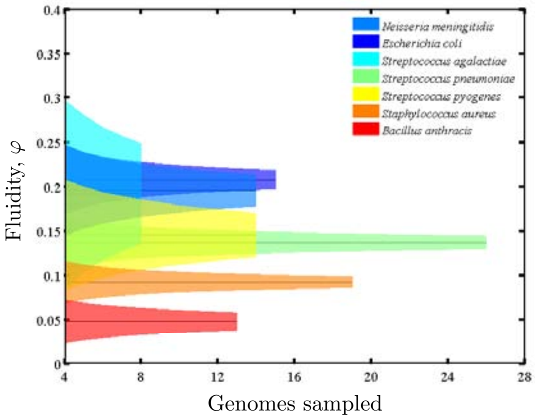

Figure S3: Convergence of mean fluidity and its variance with increases in the number of sampled genomes. Fluidity was calculated as described in the text given alignment parameters $i = 0.50$ and $c = 0.50$. The variance of fluidity is estimated as a total variance, containing both the variance due to subsampling within the sample of genomes, and the variance due to the limited number of sampled genomes.

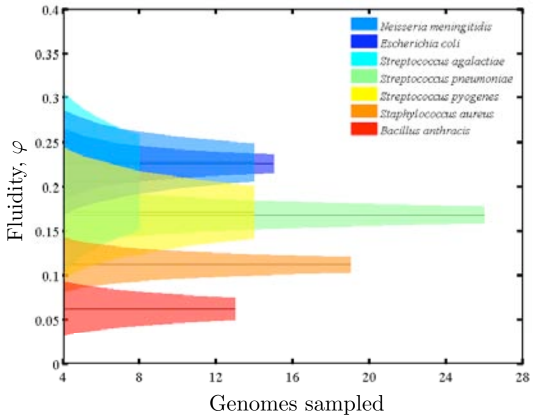

Figure S4: Convergence of mean fluidity and its variance with increases in the number of sampled genomes. Fluidity was calculated as described in the text given alignment parameters $i = 0.62$ and $c = 0.62$. The variance of fluidity is estimated as a total variance, containing both the variance due to subsampling within the sample of genomes, and the variance due to the limited number of sampled genomes.

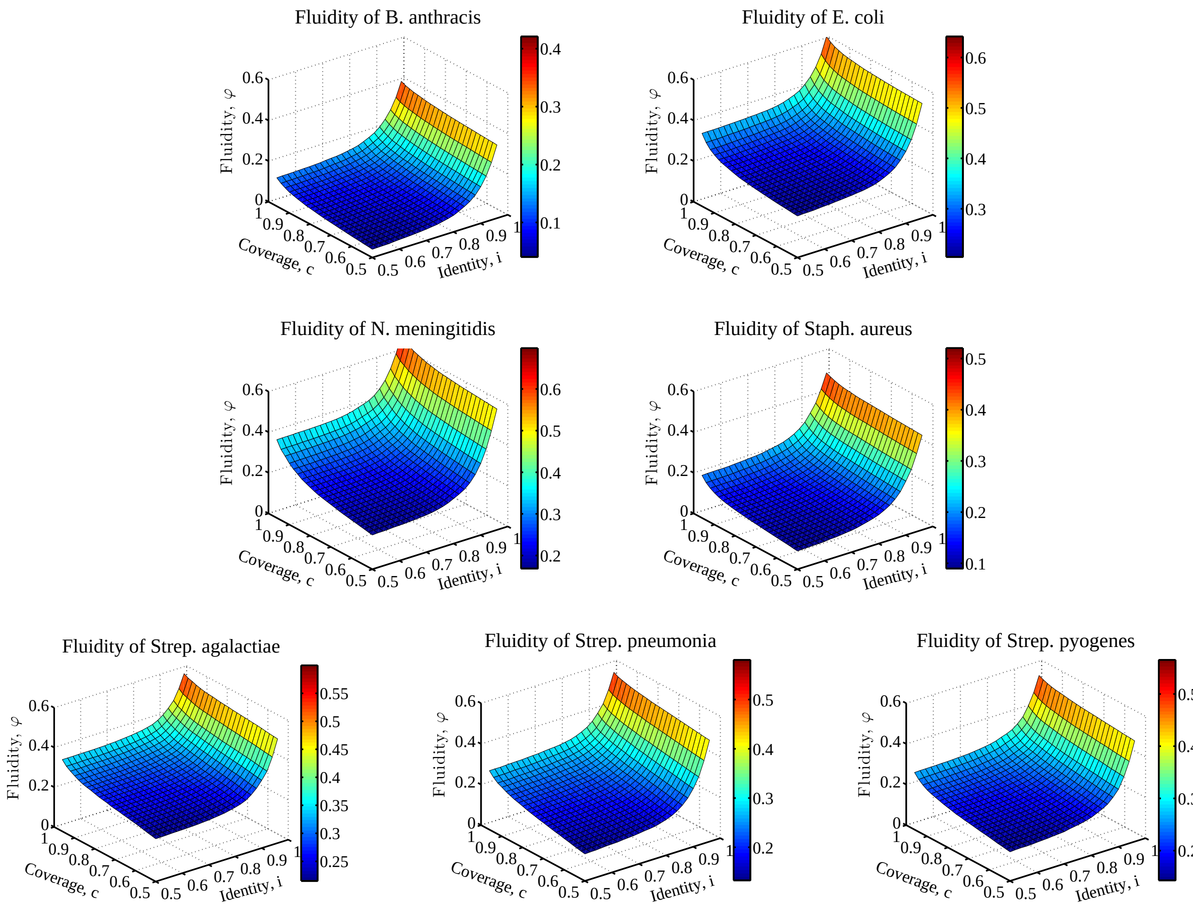

Figure S5: Estimates of fluidity depend on gene alignment parameters that determine the grouping of genes into gene families. We calculated fluidity for each of the 7 species examined in the main text with varying alignment parameter levels of identity (i) and coverage (c). We chose levels such that $0.5 \leq i \leq 0.96$ and $0.5 \leq c \leq 0.96$. Computations of φ are based on estimating the fraction of unique genes between any two random genomes. Unsurprisingly, fluidity increases with increases in either i or c. This increase arises because greater stringency of alignment causes the bioinformatics pipeline algorithm to infer that there are more unique genes. For each of the 7 species examined, genomic fluidity is more sensitive to changes in identity than to changes in coverage. This result suggests the importance of considering the robustness of results derived from bioinformatics pipelines to changes in parameters. Despite the change in fluidity values, the actual value of fluidity is relatively insensitive to changes in alignment parameters so long as neither parameter is greater than approximately 0.8. Hence, in the main text we restrict sensitivity analyses to $0.5 \leq i < 0.8$ and $0.5 \leq c < 0.8$.

References

[1] Delcher, A. L., Harmon, D., Kasif, S., White, O. & Salzberg, S. L. Improved microbial gene identification with GLIMMER. *Nucleic Acids Research* **27**, 4636–4641 (1999).

[2] Besemer, J., Lomsadze, A. & Borodovsky, M. GeneMarkS: a self-training method for prediction of gene starts in microbial genomes. Implications for finding sequence motifs in regulatory regions. *Nucleic Acids Res* **29**, 2607–2618 (2001).

[3] Altschul, S. F. *et al.* Gapped BLAST and PSI-BLAST: a new generation of protein database search programs. *Nucleic Acids Research* **25**, 3389–3402 (1997).
